# Pathophysiology of RAGE in inflammatory diseases

**DOI:** 10.3389/fimmu.2022.931473

**Published:** 2022-07-29

**Authors:** Hanbing Dong, Yue Zhang, Yu Huang, Hui Deng

**Affiliations:** Department of Neurology and Neuroscience Center, The First Hospital of Jilin University, Changchun, China

**Keywords:** RAGE, ligands, signaling, inflammatory diseases, RAGE inhibitors

## Abstract

The receptor for advanced glycation end products (RAGE) is a non-specific multi-ligand pattern recognition receptor capable of binding to a range of structurally diverse ligands, expressed on a variety of cell types, and performing different functions. The ligand-RAGE axis can trigger a range of signaling events that are associated with diabetes and its complications, neurological disorders, cancer, inflammation and other diseases. Since RAGE is involved in the pathophysiological processes of many diseases, targeting RAGE may be an effective strategy to block RAGE signaling.

## 1 Introduction

The receptor for advanced glycation end products (RAGE) is a multi-ligand receptor, a member of the immunoglobulin (Ig) superfamily, whose structure can be divided into three parts: the extracellular segment, the transmembrane segment and the intracellular segment. The extracellular segment of RAGE is the ligand binding site and consists of three immunoglobulin domains, including the V-type, C1-type and C2-type domains (1). The extracellular segment is followed by a transmembrane segment and a short, highly charged intracellular segment, which is primarily associated with signaling (2, 3). The cytoplasmic tail region of RAGE (ctRAGE) binds to diaphanous-related formin 1 (DIAPH+1), toll-interleukin 1 receptor domain containing adaptor protein (TIRAP) and other bridging proteins, which in turn activate downstream signaling (4–6). RAGE is expressed on a variety of cell types, such as vascular endothelial cells, immune cells, monocytes/macrophages, neurons, cardiomyocytes, adipocytes, glomerular epithelial cells, podocytes and alveolar epithelial cells (7–11).

The human RAGE gene is located on the major histocompatibility complex locus in the class III region of chromosome 6p21.3, a gene-dense region containing many inflammatory genes (12). To date, multiple genetic variant forms of the RAGE gene have been identified, including -429T/C (rs1800625), -374T/A (rs1800624), and G82S (rs2070600), which may affect RAGE expression or function. The RAGE -429 T/C and -374 T/A genotypes have been shown to have an effect on transcriptional activity (13). The RAGE G82S genotype upregulates the binding of S100 proteins to RAGE (14).

In addition to cell surface RAGE, there are two known forms of soluble RAGE. The first type is sRAGE, which consists of extracellular ligand-binding structural domains that are proteolytically cleaved by matrix metalloproteinases (MMPs), integrin α(ITGα), and metalloproteinase (ADAM)-10 pairs of full-length RAGE bound to the cell membrane (flRAGE). The second type is endogenously secreted RAGE (esRAGE), which is a splice variant that is actively secreted by the cell. sRAGE and esRAGE both bind RAGE ligands, preventing them from interacting with flRAGE and acting as ligand inhibitors in humans (15, 16).

Cell surface RAGE and its ligand interactions are involved in cellular cascade responses leading to inflammatory phenotypes *in vitro* and *in vivo*, and are involved in the pathophysiology of a variety of diseases. The function of nuclear RAGE has recently been explored and it was found that, unlike the function of surface RAGE, nuclear RAGE may be essential for DNA damage repair, the mechanism of which and the cell types involved are unclear (17, 18). It follows that RAGE has a dual function: as a cell surface receptor, it is part of the pro-inflammatory response, and in the nucleus, it is part of DNA repair. This review focuses on cell surface RAGE and its involvement in the inflammatory response.

## 2 RAGE Ligands

RAGE recognizes a variety of ligands, including endogenous and food-derived advanced glycation end products (AGEs), high mobility group box 1(HMGB1), S100 proteins, lysophosphatidic acid(LPA), amyloid beta(Aβ), phosphatidylserine(PS), complement protein C1q, islet amyloid polypeptide (IAPP),etc (1, 19–24).

### 2.1 AGEs

RAGE was first isolated from bovine lung in 1992 and named for its ability to act as a receptor for AGEs (25). AGEs are a group of irreversible products produced by the non-enzymatic glycosylation and oxidation of proteins, nucleic acids and lipids, a process known as the Maillard reaction (26). AGEs have two sources in the body, one is the synthesis of excess sugar and protein in the body, and the other is exogenous intake, mainly dietary intake, and AGEs have also been found in cigarette smoke (27). Methylglyoxal(MGO), the main precursor of AGEs, is a highly reactive dicarbonyl compound formed mainly as a by-product of glycolysis. Under physiological conditions, MGO is detoxified to D-lactate by the glyoxalase system, and glyoxalase I(GLO1) serves as the key enzyme for this process (28). More than 20 different AGEs have been identified in human blood and tissues as well as in food, including carboxymethyl lysine (CML), carboxyethyl lysine(CEL), pyrroline, pentosidine and methylglyoxal-lysine dimer(MOLD) (29, 30). Many studies have reported an association between AGEs and various chronic inflammatory diseases, and the accumulation of AGEs *in vivo* is associated with various pathophysiological conditions such as diabetes (31),cardiovascular diseases (32), neurological diseases (33) and cancer (34). AGEs induce pathological processes through three main mechanisms: first, by altering the structural features of proteins through the formation of crosslinks; second, by interacting with their receptor RAGE, which activates downstream signaling pathways leading to increased production of reactive oxygen species and inflammatory cytokines; and third, by intracellular accumulation (35).

### 2.2 HMGB1

HMGB1 was the first non-AGEs ligand identified to bind to RAGE (36). It was first extracted from calf thymus chromatin in 1973 and was named for its high mobility in gel electrophoresis (37). HMGB1 is an evolutionarily conserved nuclear protein consisting of three functional regions: two N-terminal DNA-binding structural domains (A-box and B-box) and an acidic C-terminal structural domain, of which the HMGB1 B-box is associated with the release of cytokines and is the functional structural domain that elicits the inflammatory response (38). HMGB1 is widely distributed in brain, heart, lung, liver, spleen, kidney, lymph and other tissues, and can be detected in the nucleus, cytoplasm and extracellular (39). In different parts of the cell, HMGB1 has different functions. In the nucleus, HMGB1 acts as a DNA chaperone, participating in DNA repair and maintaining chromosome stability; in the cytoplasm, HMGB1 interacts with Beclin-1 to induce autophagy; in the extracellular, HMGB1 is associated with inflammatory responses and immunosuppression (40). HMGB1 is mainly located in the nucleus at rest, however, under conditions of cell activation, stress, and injury, HMGB1 can enter the cytoplasm through post-translational modifications such as acetylation, poly ADP-ribosylation, and eventually be released into the extracellular space during cell activation and apoptosis (40–42). Extracellular HMGB1 can interact with RAGE, Toll-like receptors and receptors for cytoplasmic DNA/RNA sensors mediating inflammation to promote immune cell maturation, activation and cytokine production and thus participate in a variety of pathological processes (43, 44).

### 2.3 S100 proteins

The S100 family is one of the largest subfamilies of calcium-binding proteins, and more than 20 members of this family have been identified to date, including S100A1-S100A18, S100B, S100G, S100P and S100Z (45). They share a high degree of sequence and structural similarity, where each protein is encoded by a single gene. Of the more than 20 human S100 genes, the group A S100 protein is located within chromosome 1q21, with the other members (S100B, S100G, S100P and S100Z) mapping to different regions (46). In cells, most S100 proteins exist as homodimers, while a few form heterodimers, trimers and tetramers. They can exist as monomers under specific conditions, but the dimers exhibit important biological functions (47–49). The S100 protein family acts as a Ca2+ sensor and regulates many activities inside and outside the cell in a Ca2+ dependent manner (50). Inside cells, S100 proteins undergo conformational changes by binding to Ca2+, exposing binding sites to target proteins and producing biological effects, such as regulation of gene expression, enzyme activation, cell cycle, cytoskeleton composition, cytosolic Ca2+ concentration and inflammatory responses. Certain members of the S100 protein family are released extracellularly in an autocrine or paracrine manner and activate multiple inflammatory signaling pathways by interacting with a variety of cell surface receptors, including RAGE, G protein-coupled receptors, Toll-like receptor 4 (TLR4), scavenger receptors, and fibroblast growth factor receptor 1 (FGFR1), ultimately activating transcription of pro-inflammatory factors, including TNF-α, IL-1β, IL-6, and IL-8, as well as leading to reactive oxygen species production and apoptosis (47). The S100 proteins found to bind to RAGE include S100A1, S100A2, S100A4-9, S100A11, S100A12, S100A13, S100B and S100P (48, 51). Studies have shown that S100 protein expression is significantly altered in many tumors, neurodegenerative diseases, inflammatory and autoimmune diseases and can be used as a marker for these diseases (20, 52, 53).

### 2.4 LPA

LPA is a small, naturally occurring glycerophospholipid that consists of a glycerol backbone with an ester-linked acyl chain and a phosphate group. LPA is produced by the action of various lysophospholipases, including autotaxin(ATX) and phospholipases A1 or A2 (54, 55).Two different LPA species are known, including saturated fatty acids (16:0, 18:0) and unsaturated fatty acids (16:1, 18:1, 18:2, 20: 4). In humans, the most abundant form of LPA is 16:0-LPA, followed by 18:2-LPA and 18:1-LPA (56–59). Different LPA types are recognized by different LPA receptors and have different biological activities. LPA mediates its action mainly by activating six known G protein-coupled receptors (GPCRs) (60). In addition, LPA can bind and activate the atypical receptor RAGE (23) and the cation channel transient receptor potential vanilloid 1 (TRPV1) (61). LPA and LPA receptor signaling has been found to be associated with a variety of physiological and pathophysiological conditions, including cancer (62), cardiovascular disease (63), diabetic microvascular complications (64), neuropathic pain (65),etc. RAGE is involved in LPA-induced phosphatidylinositol 3-kinase/protein kinase B(PI3K/AKT) and activation of extracellular signal-regulated kinas (ERK), among others (23, 66, 67). Although LPA and its interaction with RAGE have been identified, the involvement of LPA-RAGE under different pathophysiological conditions remains to be further explored.

### 2.5 Aβ

Aβ is a cleaved form of amyloid precursor protein produced by hydrolytic cleavage of proteins and is a major neuropathological marker of Alzheimer’s disease(AD) (68). The two most prevalent forms of Aβ are Aβ1-40 and Aβ1-42, with Aβ1-42 being the most toxic form that accumulates in the brains of AD patients (69). It was shown that RAGE is upregulated in AD and that the V domain of RAGE binds to Aβ oligomers, while the C1 domain of RAGE interacts with Aβ aggregates and is involved in the neurotoxic effects of Aβ in the brain (70). Aβ activation of RAGE induces oxidative stress in neurons and pro-inflammatory cytokine production in microglia by stimulating NADPH oxidase production of reactive oxygen species (71, 72). In addition, RAGE in the endothelium mediates Aβ transport across the blood-brain barrier into the central nervous system, leading to Aβ accumulation in brain tissue and promoting the formation of neuroinflammatory plaques (73). Taken together, Aβ is a potent ligand for RAGE and their binding is involved in the pathophysiological process of the disease, which provides evidence that blocking this interaction may open up new ideas for RAGE-mediated therapy.

### 2.6 Phosphatidylserine

PS is a negatively charged phospholipid, which is one of the important components of cell membrane phospholipids and has an important role in the regulation of many cellular metabolic processes. PS, a negatively charged phospholipid normally confined to the inner plasma membrane leaflet by flippases, is externalized on the apoptotic cell surface by scramblases, and is an important signal for macrophage recognition and clearance of apoptotic cells (74, 75). PS initiates phagocytic signaling, allowing target cells to be recognized by a wide range of receptors located on the surface of macrophages, including brain-specific angiogenesis inhibitor 1 (BAI-1), T-cell immunoglobulin mucin 4 (TIM-4), stabilin-2, and RAGE (22, 76–78). Under apoptotic conditions, PS binds to RAGE on macrophages leading to activation of Rac1/Cdc42 and ERK and induction of phagocytosis (79). It has been shown that RAGE dysfunction is associated with abnormal alveolar epithelial remodeling occurring in the pathogenesis of pulmonary fibrosis (80).The specific structural domains of PS interaction with RAGE are currently unknown and the interaction between the two is still under investigation. The molecular mechanism of downstream signaling induced by this interaction needs to be further revealed.

### 2.7 C1q

C1q is a complement protein assembled from 18 polypeptide chains with a C-terminal spherical head region that mediates recognition of multiple molecular structures and an N-terminal collagen-like tail that mediates immune effector mechanisms (81). C1q is mainly synthesized and secreted in phagocytic cells such as macrophages, dendritic cells and microglia. In addition to its role in the classical pathway of complement activation, C1q has other important immune functions, including enhancing phagocytosis and regulating cellular functions in the adaptive immune response (82). The function of C1q is mediated by C1q receptors on the surface of effector cells. Identified C1q receptors include C1q receptor for phagocytosis enhancement(C1qRp/CD93), complement receptor 1(CR1/CD35), calreticulin(CRT/cC1qR), receptor for the globular head of C1q(gC1qR/gC1qbp/p33), CD91/LRP1/A2MR/APOER, alpha2beta1 integrin, and RAGE (24, 83–85). Different C1q receptors may function in different cells or under specific conditions to mediate and/or amplify C1q-related functions. RAGE is a C1q receptor that enhances C1q-mediated phagocytosis (24). The mechanism and therapeutic targets of C1q-RAGE binding action need to be further understood.

The extracellular segment of RAGE is the ligand binding site and consists of three immunoglobulin domains—V, C1, C2. Most reports on the structural biology of RAGE suggest that the main site of binding of different ligands is the V domain. Although some ligands appear to bind to C1 or C2 domains, much evidence suggests that the V domain is the primary site of ligand involvement. Therefore, pharmacological strategies for targeting RAGE are largely focused on this domain. **Table 1** lists a few major types of RAGE ligands and the putative domains of the receptor to which they bind.

**Table 1:** A few major types of RAGE ligands and RAGE binding domain.

Ligands	RAGE Binding Domain(s)	References
AGEs	V domain	(86)
HMGB1	V domain	(36)
S100B	V domain	(48)
S100A1	V domain	(87)
S100A5	V domain	(51)
S100A6	V,C2 domains	(88, 89)
S100A9	V domain	(90)
S100A12	V,C1 domains	(91)
S100A13	C2 domain	(92)
S100P	V domain	(93)
LPA	V,C2 domains	(23)
Aβ oligomers	V domain	(70)
Aβ aggregates	C1 domain	(70)

## 3 RAGE-mediated signaling pathways

**Figure 1:**
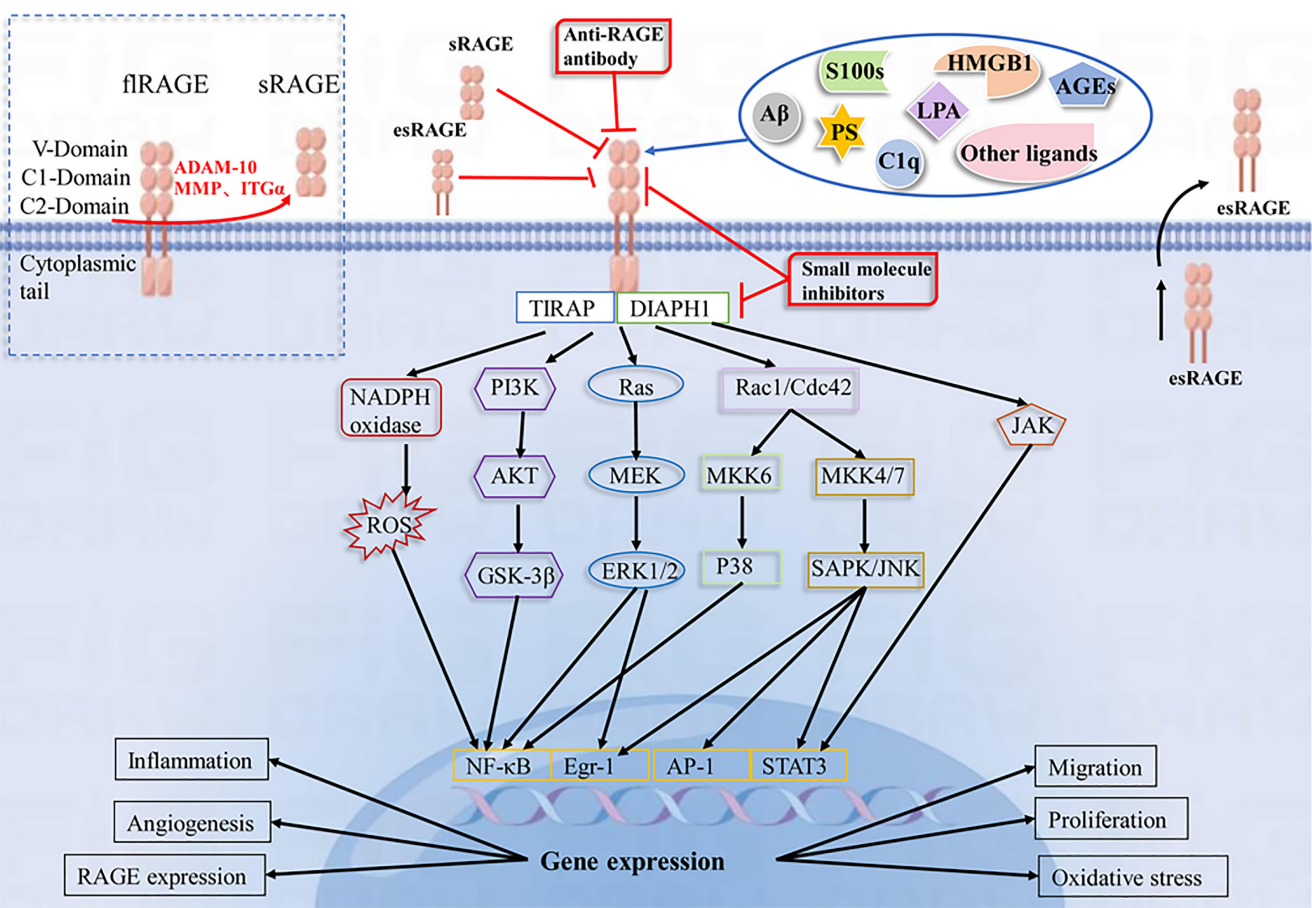
Schematic representation of the RAGE-Ligand axis and their blocking strategies. RAGE consists of three immunoglobulin domains(V, C1, C2), a transmembrane segment and a short C-terminal structural domain. RAGE recognizes a variety of ligands, including AGEs, HMGB1, S100 proteins, LPA, Aβ, PS, and C1q. The interaction of RAGE with ligands leads to activation of NADPH oxidase, PI3K/AKT, MEK/ERK, SAPK/JNK, and JAK/STAT pathways, which further activate intracellular transcription factors such as NF-κB, Egr-1, AP-1, and STAT3. This leads to changes in gene expression and alterations in cellular functions, including inflammation, oxidative stress, angiogenesis, proliferation, migration and upregulation of RAGE expression. sRAGE, esRAGE, anti-RAGE antibodies and RAGE small molecule inhibitors have a blocking effect on the RAGE pathway.

Ligand binding to RAGE has been shown to activate multiple cellular signaling cascades, including NADPH oxidase, Ras/mitogen-activated protein kinase kinase/ERK1/2 (Ras/MEK/ERK1/2), stress-activated protein kinase/c-Jun amino-terminal kinase (SAPK/JNK), mitogen-activated protein kinase (MAPK)/P38, PI3K/AKT, small GTPase Cdc42/Rac1, janus kinase/signal transducer and activator of transcription (JAK/STAT) and glycogen synthase kinase 3β (GSK-3β) pathways, leading to activation of many transcription factors, including NF-κB, STAT3, activator protein 1 (AP-1), early growth response-1 (Egr-1), resulting in increased IL-1, IL-6 and TNF-α synthesis and release, among others (94–98). In addition, DIAPH1 is required as a RAGE bridging protein for active signaling and molecular pathways in a variety of vascular and inflammatory cells, such as endothelial cells, smooth muscle cells, and macrophages (3, 99, 100). Evidence suggests that the interaction of ctRAGE with DIAPH1 is critical for intracellular signaling such as AKT phosphorylation and the JAK/STAT signaling cascade (101–103). (**Figure 1**). In a human umbilical vein endothelial cell model, DIAPH1 plays a key role in AGEs-induced increase in microvascular permeability by binding to RAGE, and blocking RAGE-DIAPH1 binding can eliminate AGEs-induced hyperpermeability in endothelial cells (100). In neurons, RAGE interacts with AGEs, HMGB1 and S100 proteins to induce activation of the NF-κB pathway, leading to neuronal cell death (7, 104, 105). Aβ binding to neuronal cell RAGE leads to neurodegeneration and neuronal cell death *via* p21ras, p38, SAPK/JNK, PI3K/AKT and JAK/STAT pathways (106, 107). In vascular endothelial cells, S100 proteins binding to RAGE activates PI3K, ERK1/2 and JNK, ultimately leading to the activation of transcription factor Egr-1, which releases IL-6, CCL-2, vascular cell adhesion molecule-1 (VCAM-1) and ICAM-1, further inducing vascular dysfunction or atherosclerosis. S100 proteins binding to RAGE can Activate NADPH oxidase, trigger intracellular ROS production, upregulate vascular smooth muscle osteogenic gene expression, and increase vascular calcification and plaque instability (20). Activation of MAPK/p38 and SAPK/JNK was observed in monocytes/macrophages and tumor cells (108–110). In hepatocytes, HMGB1 interacts with RAGE to activate the MEK1/2/ERK1/2/JNK pathway and increase type I collagen deposition, promote TGF-β production, and participate in the pathogenesis of liver fibrosis (19). These signaling cascades have been shown to lead to further synthesis of RAGE, which in turn activates more pro-inflammatory factors, thus creating a positive feedback loop to enhance the inflammatory response. In RAGE-mediated cell signaling, the diversity of signaling cascades suggests that different RAGE ligands may induce different signaling pathways (especially in different cell types), thus further increasing the complexity of the RAGE network.

## 4 RAGE and inflammatory diseases

RAGE and its ligands are associated with a variety of inflammatory diseases and are a major inflammatory driver in their pathogenesis.

### 4.1 RAGE and diabetes complications

Diabetes is a chronic metabolic disease characterized by hyperglycemia, whose high blood glucose levels lead to the accumulation of AGEs, which accelerates the development of vascular complications, including diabetic nephropathy, retinopathy, peripheral neuropathy and cardiovascular disease (111, 112).

The major microvascular complications of diabetes affect the kidney, retina and peripheral nerves. In studies related to diabetic nephropathy, interaction between AGEs and RAGE on glomerular foot cells increases the expression of acetyl heparinase, which degrades acetyl heparan sulfate in the glomerular basement membrane (GBM) and disrupts the filtration barrier, by activating the NF-κB signaling pathway and plays an important role in diabetic nephropathy. Compared with wild-type mice, RAGE-deficient diabetic mice exhibit slower progression of diabetic nephropathy, less expression of inflammatory and fibrotic mediators in renal tissues, and greater resistance to renal cell apoptosis (113). In addition, DIAPH1-deficient diabetic mice also exhibited a protective effect on diabetic kidneys compared to diabetic mice expressing DIAPH1, suggesting that DIAPH1 is involved in the pathophysiological processes of diabetic nephropathy (114). In studies related to diabetic retinopathy, RAGE and its ligands AGEs, S100 proteins and HMGB1 were found to be up-regulated in the vitreous and preretinal membranes with proliferative diabetic retinopathy and proliferative vitreoretinal retinopathy (115). HMGB1 interacts with Toll-like receptor 4 (TLR4) and RAGE and activates NF-κB to generate an inflammatory response and disrupt the retinal vascular barrier (116). In diabetic neuropathy, electrophysiological and morphological changes within peripheral nerves and dorsal root ganglia are attenuated in RAGE-deficient diabetic mice compared to wild-type mice. In addition, increased expression of HMGB1 and RAGE in diabetic peripheral neuropathy (117). The interaction analysis using GeneMANIA software suggests that HMGB1-RAGE-DIAPH1 interaction may be critical for the progression of diabetic peripheral neuropathy (103).

Cardiovascular disease is a macrovascular complication of diabetes and a major cause of death in diabetes, and its pathophysiology is the narrowing of the arterial wall due to atherosclerotic lesions. In experiments to validate the role of RAGE in diabetic atherosclerosis, total atherosclerotic plaque area was found to be significantly increased in apolipoprotein E (apoE) knockout diabetic mice compared with non-diabetic apoE -/- mice, whereas plaque accumulation in apoE and RAGE double knockout diabetic mice was not significantly different from non-diabetic apoE -/- mice. In addition, plaque area was further reduced in apoE and RAGE double knockout non-diabetic mice compared with non-diabetic apoE -/- mice, confirming the protective effect of RAGE knockout on the development of atherosclerosis (118). Another study confirmed that sRAGE treatment significantly reduced the size and complexity of atherosclerotic lesions, while a decrease in the expression of pro-inflammatory mediators such as inflammatory cell chemokines (JE-MCP-1 and VCAM-1), cox-2, and tissue factor was observed (119). Burke et al. performed autopsy and immunohistochemical staining of coronary arteries from diabetic and non-diabetic subjects with sudden cardiac death and found that macrophage plaque area and T-cell infiltration were significantly higher in diabetic subjects than in non-diabetic patients. In addition, RAGE and its ligand S100A12 were expressed in the atherosclerotic plaques of both groups of subjects, but to a significantly higher extent in diabetic than in non-diabetic subjects (120). Mac-1/RAGE interaction mediates the adhesion of leukocytes to endothelial cells, and RAGE is directly involved in leukocyte migration across the vessel wall, promoting inflammatory processes and vascular remodeling, especially accelerating the atherosclerotic process in the diabetic vessel wall (121).

The effects of diabetes on the central nervous system have received increasing attention in recent years. Extensive evidence collected from rodent models of diabetes suggests the presence of electrophysiological abnormalities in the hippocampus and hippocampus-dependent behavioral abnormalities, particularly those related to learning, memory, and cognitive function (122, 123). RAGE has received significant attention in the search for potential mechanisms of diabetes-induced cognitive decompensation. An animal study showed that mice with long-term diabetes (18 to 33 weeks of diabetes) exhibited increased expression of RAGE in neurons and glial cells and showed cognitive dysfunction (124). Streptozotocin-induced wild-type diabetic mice exhibited impaired hippocampal-dependent spatial memory, whereas spatial memory in RAGE knockout diabetic mice was not significantly different from controls, further suggesting that parameters associated with hippocampal-dependent spatial memory are dependent on RAGE expression (125).

### 4.2 RAGE and neurological diseases

RAGE expression is elevated in many inflammatory neurological diseases, including Alzheimer’s disease, Parkinson’s disease, multiple sclerosis, myasthenia gravis, and cerebrovascular disease.

#### 4.2.1 RAGE and Alzheimer’s disease

Alzheimer’s disease(AD) is a degenerative disease of the central nervous system characterized by progressive cognitive dysfunction and behavioral impairment, and is the leading cause of dementia (68).

One of the pathological features of AD is the presence of a large number of neuroinflammatory plaques in the cerebral cortex, hippocampus, certain subcortical nuclei and thalamus, with Aβ being the main component of neuroinflammatory plaques. In addition, the presence of AGEs was localized in neuroinflammatory plaques (126, 127). Both Aβ and AGEs are able to bind and activate RAGE signaling and induce the expression of pro-inflammatory cytokines such as TNF-α, IL-6 and macrophage colony-stimulating factor (M-CSF) through a nuclear factor KB (NF-KB)-dependent pathway (128). It was found that RAGE expression was elevated in AD patients and AD transgenic mice (129–132). High expression of RAGE in neurons or microglia of AD mice accelerates the accumulation of Aβ and the formation of neuroinflammatory plaques and exacerbates spatial learning deficits, memory impairment, and neuropathological and biochemical changes (133, 134). Interaction of RAGE with AGEs enhances oxidative stress and exacerbates synaptic dysfunction (135). Deletion of the RAGE gene or blockade of RAGE signaling in neurons or microglia of AD mice attenuates Aβ-induced deterioration, protects spatial learning and memory abilities, and prevents synaptic dysfunction (130, 135, 136). Therefore, blocking AGEs-RAGE interactions may prevent neuroinflammation, synaptic and neuronal dysfunction, and cognitive impairment caused by AGEs accumulation and RAGE-dependent signaling. In addition, the expression of DIAPH1, an articulatory protein of ctRAGE, was also evaluated in AD patients. DIAPH1 expression was significantly increased in microglia of AD compared with age-matched controls and multiple cell types, and DIAPH1 expression was associated with increased lipid staining and inflammatory morphology (137). sRAGE may act as a decoy for RAGE and provide a negative regulatory mechanism. Plasma sRAGE levels were measured in a cross-sectional study of 152 patients with a clinical diagnosis of AD, 91 patients with vascular dementia, and 161 nondemented controls. It was shown that plasma levels of sRAGE were significantly lower in AD patients compared to vascular dementia patients or non-demented controls (138).

Neurogenic fibrillary tangles are the second major pathological hallmark of AD and are characterized by tau protein hyperphosphorylation. tau protein phosphorylation is regulated by protein kinases and protein phosphatases (PP) (139, 140). Several protein kinases, such as GSK-3, calmodulin-dependent protein kinase II(CaMKII), and MAPK family members, can phosphorylate tau proteins and are regulated by the AGEs/RAGE axis. Methylglyoxal-derived AGEs induce tau hyperphosphorylation and impair synapses and memory through RAGE-mediated GSK-3 activation, and targeted inhibition of the RAGE/GSK-3 pathway is effective in ameliorating AD-like histopathological changes and memory deterioration (141). Protein phosphatase 2A (PP2A) is the most active enzyme for dephosphorylating hyperphosphorylated tau proteins (142). *In vivo* PP2A activity is regulated by a protein called inhibitor 2 (I2PP2A). I2PP2A is cleaved by asparagine endopeptidase (AEP) into active fragments, N-terminal (I2NTF) and C-terminal (I2CTF), which bind to PP2A resulting in its inactivation. Thus, elevated AEP expression is associated with reduced PP2A activity, leading to tau protein hyperphosphorylation and highlighting its potential role in AD pathology (143). It was found that the AGEs-RAGE axis could lead to tau protein hyperphosphorylation by increasing the expression of AEP (144).

#### 4.2.2 RAGE and Parkinson’s disease

Parkinson’s disease (PD) is a neurodegenerative disorder commonly seen in middle-aged and older adults, clinically characterized by resting tremor, bradykinesia, myotonia, and postural balance disturbances. Pathological features of PD include progressive death of nigrostriatal dopaminergic neurons and accumulation of alpha-synuclein-containing Lewy bodies and Lewy synapses in degenerating neurons. The exact etiology of PD remains unclear. There is growing evidence that oxidative stress and inflammatory responses involved in the RAGE process play a crucial role in PD disease progression (105, 145).

It was shown that RAGE was strongly expressed in the substantia nigra and frontal cortex of PD patients as well as in 1-methyl-4-phenyl-1,2,3,6-tetrahydropyridine (MPTP)-induced PD mouse model (146–148). RAGE is a multi-ligand receptor of the immunoglobulin superfamily, expressed mainly by neurons and microglia, and mediates inflammatory responses by activating multiple signaling pathways. α-synuclein is a central player in causing neuronal cell death in PD, acquiring toxicity mainly through its misfolding or aggregation. It was found that α-synuclein was glycosylated in both PD models and brain tissue of PD patients, and that glycosylation induced α-synuclein oligomerization and stabilization as oligomers, exacerbating its toxic effects (149). Glycosylated α-synuclein and its oligomers activate microglia and induce the release of cytokines and NF-kB signaling proteins by interacting with RAGE, thereby activating signaling cascades in the brain and destroying an increasing number of neuronal cells (150). Furthermore, in a study of sporadic PD in a group of Chinese Han Chinese population, RAGE -429T/C gene polymorphism was found to be possibly associated with susceptibility to PD (151), suggesting that RAGE may play an important role in the pathogenesis of PD.

The study also found increased expression of S100B and HMGB1 in PD. At pro-inflammatory concentrations, S100B can interact with RAGE to activate the Ras/Rac1-Cdc42/NF-κB and Ras/MEK/ERK1/2/NF-κB pathways to induce microglia activation and release of CCL3, CCL5 and CXCL12 chemokines, exacerbating the inflammatory response (152). HMGB1, one of the RAGE ligands, can also bind to it to produce pathogenic effects (147, 153). In addition, RAGE-deficient mice treated with MPTP had a higher number of surviving dopaminergic neurons compared to their littermate control mice. A recent animal experiment applied lentiviral transfection to silence RAGE expression for the first time to investigate the role of the RAGE signaling pathway in PD, further validating the involvement of RAGE in pro-inflammatory changes during PD in animal models.The results also showed that MAPK/p38 activation mediated the secretion of RAGE-NF-κB-dependent pro-inflammatory cytokines, which induced dopamine degradation in the striatum and loss of tyrosine hydroxylase neurons in the substantia nigra. Reduction of NF-κB translocation to the nucleus by RAGE ablation, accompanied by downregulation of COX2 expression, one of the pro-inflammatory cytokines, ameliorates the RAGE-induced inflammatory response (154).

#### 4.2.3 RAGE and Myasthenia Gravis

Myasthenia gravis (MG) is an antibody-mediated autoimmune inflammatory disease of the neuromuscular junction, characterized by fluctuating skeletal muscle weakness and, in severe cases, life-threatening involvement of respiratory muscles. T cells, B cells, complement and cytokines play a key role in the pathogenic inflammation of MG (155).

The role of RAGE and its ligand S100B has been demonstrated in rats with experimental autoimmune myasthenia gravis (EAMG). In this study, an EAMG model was constructed by immunizing female Lewis rats with an AChR epitope corresponding to amino acids 97-116 of the rat α-subunit. At the late stage of initial immunization, the expression level of RAGE was significantly higher in CD4+ T cells harvested from lymph node mononuclear cells in EAMG rats compared to controls. Furthermore, based on immunohistochemical staining results, RAGE expression was elevated in the spleen of EAMG rats compared to controls, and it was confirmed that activation of RAGE exacerbates EAMG. Administration of RAGE-blocking RAGE-Fc significantly reduced disease severity in EAMG. In addition, serum levels of S100B were elevated in EAMG rats at late initial immunization (45 days) compared to controls and stimulated CD4+ T cell distribution during the EAMG process, and in addition, the interaction of RAGE with S100B upregulated AChR-specific T cell proliferation. The findings suggest that RAGE and its ligand S100B play an important role in EAMG progression by regulating T cell responses and inducing splenocyte-derived AChR-specific antibody production (156). In another study on EAMG rats, EAMG rats with diabetes mellitus showed more symptoms of muscle weakness and poorer clinical scores compared to controls by an unclear mechanism, which was considered to be related to elevated levels of AGEs caused by hyperglycemia, which triggers inflammation by binding to specific receptors, and AGEs induced maturation of dendritic cells (DCs) and enhanced their ability to stimulate T cell proliferation and cytokine production (157).

Moser et al. conducted a cross-sectional study on the role of the RAGE pathway in the pathophysiology of MG, including 42 MG patients and 36 healthy controls (158). It was shown that the levels of soluble receptors sRAGE and esRAGE were significantly lower in MG patients compared to controls, especially in late onset MG (LOMG), and that sRAGE levels did not correlate significantly with drug therapy, clinical manifestations of the disease, or the presence of AChR-specific antibodies. In the same study, correlations were performed for the RAGE ligands HMGB1, S100B, S100A8, and AGE-CML, with no significant differences compared to controls. These results suggest that RAGE may also play a novel role in the pathogenesis and progression of human MG.

The thymus is the site of T cell differentiation, development and maturation, and its role in the pathogenesis of MG is well established. Thymus abnormalities, including thymoma and thymic hyperplasia, are present in about 80% of MG patients. Thymoma is found in about 20-30% of MG patients, and MG is present in about 30-50% of thymoma patients (159). The abnormal immune response in MG patients is closely related to the thymus. Moser et al. investigated the role of RAGE in thymic abnormalities (160) and reported a strong accumulation of RAGE and its ligand HMGB1 in all histological types of thymic epithelial tumors, which was particularly significant in the most aggressive types - thymic carcinoma and B3 thymoma. RAGE expression was elevated in thymocytes, macrophages, Hassall vesicles, thymic medulla and germinal center cells in patients with myasthenia gravis, suggesting that the RAGE pathway may have an impact on the pathogenesis of myasthenia gravis. In another study, thymus specimens from 41 patients with myasthenia gravis (18 thymomas, 17 hyperplasia and 6 other types of pathology) were subjected to RAGE using immunohistochemical methods (161). In agreement with Moser’s findings, RAGE was more significantly expressed in MG combined with thymoma, especially type B2. All of the above findings suggest that the RAGE pathway is involved in the pathophysiological processes of MG, and is more pronounced in MG with thymic abnormalities.

#### 4.2.4 RAGE and Multiple Sclerosis

Multiple sclerosis (MS) is a demyelinating autoimmune disease of the central nervous system. The innate immune system, composed of CNS-resident microglia and infiltrating macrophages, and the adaptive immune system, composed of T lymphocytes and B lymphocytes, play key roles (162).

Previous studies have shown that RAGE and its ligand HMGB1 are highly expressed in active lesions of MS and its corresponding animal model of experimental autoimmune encephalomyelitis (EAE), in which microglia and macrophages are the main sources of HMGB1 in MS and EAE lesions, and that HMGB1 levels correlate with active inflammation. The interaction of HMGB1 with RAGE not only induces the secretion of pro-inflammatory cytokines but also mediates the upregulation of cell adhesion molecules (ICAM-1, VCAM-1 and E-selectin) expression, amplifying the inflammatory response in the pathogenesis of MS and EAE (163, 164).

Immune cells are infiltrated and activated in the CNS, and the metabolic pattern changes from oxidative phosphorylation (OXPHOS) to glycolysis (165, 166). A glycation agent, MGO, is produced during glycolysis, which in turn is derived into AGEs that induce an inflammatory response by activating the RAGE pathway. Compared with healthy controls, serum levels of AGEs were increased and sRAGE levels were significantly lower in MS patients, and sRAGE serum levels were negatively correlated with the Expanded Disability Status Scale (EDSS) and clinical relapse rates (167). Similarly, esRAGE serum levels correlated with the Multiple Sclerosis Severity Scale (MSSS) and six-month clinical relapse rates. In addition, serum esRAGE levels were significantly higher in MS patients treated with disease-modifying drugs(DMDs) compared with those not treated with DMDs (168). Sternberg et al. found a significant increase in the percentage of RAGE-positive monocytes and T lymphocytes in the peripheral blood of MS patients (169),further supporting the involvement of RAGE in MS pathophysiological processes.

The relevance of RAGE gene polymorphisms in MS patients has been described. In a study of a Hungarian population, significant differences in the distribution of RAGE -374 T/A genotypes were found between controls and MS patients, while no differences were observed between MS patients and controls in the distribution of RAGE -479 T/C and G82S genotypes (170). However, in a Chinese population study, the RAGE G82S gene polymorphism was found to be significantly different in MS patients and healthy controls (171). Although these two studies revealed differences in RAGE gene polymorphisms in MS patients compared to controls, genome-wide association studies were unable to confirm these polymorphisms in a large cohort. These results suggest that RAGE gene polymorphisms may be associated with population differences due to ethnic background, environmental factors, etc.

#### 4.2.5 RAGE and Stroke

Stroke is an acute cerebrovascular disease. It is divided into hemorrhagic stroke and ischemic stroke. Hemorrhagic strokes include cerebral hemorrhage (ICH) and subarachnoid hemorrhage (SAH). Inflammatory cells and immune cells are key factors in post-stroke injury. Stroke not only activates inflammatory and immune cells in the central nervous system, but also induces infiltration and accumulation of inflammatory and immune cells in the peripheral system. Both glial cells and peripheral immune cells are involved in regulating the inflammatory response to stroke (172).

RAGE and its ligand HMGB1 are among the factors involved in the inflammatory response in stroke. Elevated levels of HMGB1 were found in the serum of ischemic stroke patients and in the brain tissue of ischemic mice, and inhibition of HMGB1 improved ischemic brain injury in mice (173). *In vitro* studies showed that RAGE expression in glial cells mediated the toxic effects of HMGB1 (173). Another study found that in a mouse model of ischemic stroke, hyperglycemia increased infarct volume and decreased the number of protective noninflammatory monocytes/macrophages in the ischemic brain compared with controls. This process is mainly mediated by α-dicarbonyl (AGEs precursors) and RAGE (174). In a study on the correlation between early brain injury caused by ICH and RAGE pathways, it was found that RAGE and its ligand HMGB1 expression was increased 12 hours after ICH, along with increased NF-κB p65 expression, increased blood-brain barrier permeability, brain edema, motor dysfunction and nerve fiber damage. In addition, local levels of inflammatory factors IL-1β, IL-6, and IL-8R were also elevated. Inhibition of RAGE expression improved these conditions (175). In conclusion, these findings highlight the role of ligand-RAGE signaling in the pathophysiology of ICH. However, the presence of RAGE is not always detrimental. It was shown that RAGE was upregulated in SAH rats and that the increased RAGE was mainly expressed by neurons and microglia (176). In the treatment of SAH rats with RAGE inhibitors, it was found that inhibition of RAGE significantly reduced brain edema and improved neurological function one day after SAH. However, at three days after SAH, RAGE inhibitor-treated rats exhibited increased neuronal cell death, higher levels of apoptosis and reduced autophagy. These data suggest that, at least in the rat model of SAH, RAGE may exert both destructive and protective effects. This may be based on its role in different cell types and the timing of the post-SAH response (177).

In conclusion, RAGE and its ligands are involved in the inflammatory response to stroke. Blocking effects on the RAGE axis are not always beneficial or detrimental, which emphasizes the possibility that RAGE exerts different effects depending on the cell type acting and the period of stroke. More extensive time-course studies of RAGE and its ligands and further exploration of their role in stroke patients are necessary to provide new ideas for the treatment of stroke.

### 4.3 RAGE and Cancer

The RAGE signaling pathway is involved in the development of a variety of cancers, including glioma, bladder cancer, breast cancer, melanoma, liver cancer, pancreatic cancer, prostate cancer, colorectal cancer, ovarian cancer, gastric cancer, lung cancer,etc (178–185). Malignant tumor cells as well as multiple cell types within the tumor microenvironment (including fibroblasts, leukocytes and vascular cells) can express and secrete a variety of RAGE ligands. These ligands act synergistically in an autocrine and paracrine manner to control different forms of cellular signaling, including inflammation, proliferation, apoptosis, autophagy and migration, to promote malignant progression of cancer (186, 187). Gene expression analysis of human patients and mouse tumor models revealed overexpression of RAGE and its ligands in a variety of solid tumors. RAGE interferes with apoptosis *via* a p53-dependent mitochondrial pathway and controls autophagy by decreasing phosphorylation of mammalian target of rapamycin (mTOR), anti-apoptotic proteins, and increasing Beclin-1/VPS34 autophagosome formation (188). Blocking RAGE signaling slows cancer invasion and metastasis in cellular and animal models (3, 110, 179, 189).

RAGE signaling can also influence the malignancy of tumors by acting on the tumor microenvironment. Hypoxia is a prominent feature of the tumor microenvironment. Hypoxia induces RAGE expression in tumor cells, promotes leukocyte recruitment and increases inflammatory stress in the tumor microenvironment, which further enhances tumor cell proliferation, angiogenesis and metastasis (178). Myeloid-derived suppressor cells (MDSC) are an important component of the tumor microenvironment and have the ability to significantly suppress the immune cell response. They originate from common myeloid primitive cells in the bone marrow, accumulate in response to multiple pro-inflammatory mediators, and are recruited to the tumor microenvironment by chemokines.RAGE contributes to MDSC production and enhances their function (190, 191). Thus, RAGE affects not only tumor cells but also the tumor microenvironment, which is essential for tumor growth, invasion and metastasis. It is a highly researched therapeutic target in cancer.

### 4.4 RAGE and other inflammatory diseases

**Figure 2:**
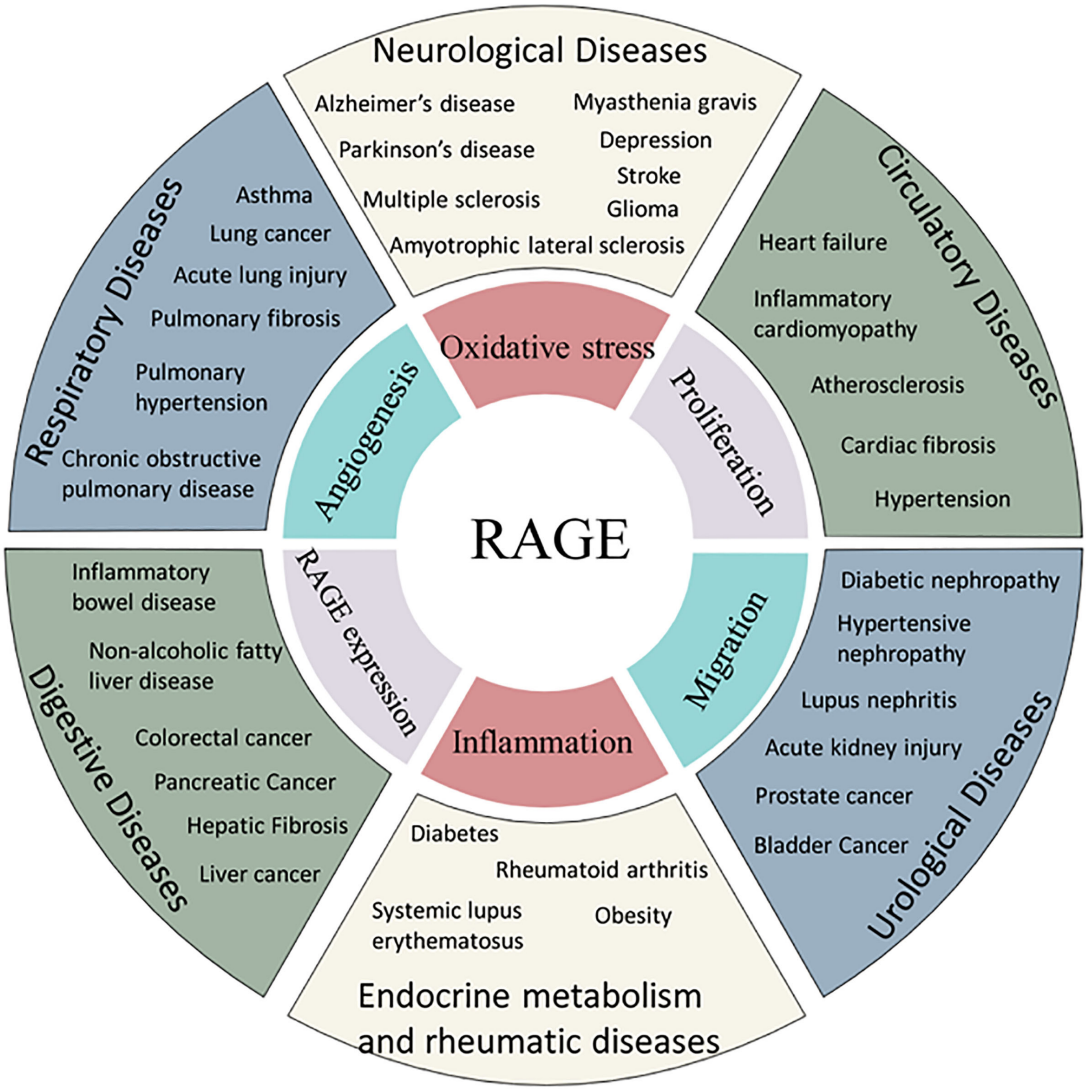
Association of RAGE and human diseases. AGEs are related to the incidence of several diseases through common mechanism of oxidative stress, angiogenesis, proliferation, inflammation or migration.

The RAGE axis is involved in rheumatoid arthritis (192), systemic lupus erythematosus (193), amyotrophic lateral sclerosis (194), inflammatory bowel disease (195), depression (196) and other diseases. In addition, RAGE is involved in the fibrotic process in several organs, such as hepatic fibrosis (19), cardiac fibrosis (197), pulmonary fibrosis (198) and renal fibrosis (199) (**Figure 2**).

## 5 RAGE Inhibitors

Research on RAGE as a target for disease therapy is currently focused on sRAGE, anti-RAGE antibodies and RAGE small molecule inhibitors. According to the difference of RAGE small molecule inhibitors targeting the RAGE region, they are divided into two categories: inhibitors targeting the extracellular segment of RAGE and inhibitors targeting the intracellular segment of RAGE.

### 5.1 Targeted RAGE extracellular segment inhibitors

#### 5.1.1 TTP488

TTP488,also known as PF-04494700 or azeliragon, is an orally administered small molecule RAGE inhibitor. Preclinical evidence suggests that TTP488 blocks the binding of RAGE to AGEs, HMGB1, S100 proteins and Aβ and improves cognitive function (200). In the clinical phase III trial of TTP488 for AD, there was no significant improvement in cognition or function in patients taking TTP488 compared to placebo, and the clinical drug trial ended in failure. However, as a typical RAGE extracellular segment inhibitor, the application of TTP488 in other related diseases and the structural modification and conformational relationship studies based on the existing ones are still valuable to be explored.

#### 5.1.2 FPS-ZM1

FPS-ZM1 is a specific RAGE inhibitor screened from more than 5000 compounds with tertiary amide as the basic pharmacodynamic group. FPS-ZM1 can cross the blood-brain barrier and block the binding of Aβ to RAGE by binding to the V domain of RAGE without interfering with the binding of Aβ to other receptors (201). FPS-ZM1 has been reported to play a potential therapeutic role in a variety of disease models, such as neurodegeneration (202), diabetes (203), cardiovascular disease (204) and cancer (179). In primary cultured rat microglia, FPS-ZM1 significantly inhibits AGEs-induced inflammation and oxidative stress (202). In an *in vitro* model of murine heart tissue (ECT), FPS-ZM1 partially attenuated the increase in ROS and inflammatory response after prolonged AGEs exposure and rescued ECT function (203). FPS-ZM1 reverses amyloid Medin-induced endothelial dysfunction and oxidative stress in a human umbilical vein endothelial cell model (204). *In vitro* studies on highly metastatic breast cancer cells showed that FPS-ZM1 eliminated the over-invasion caused by RAGE. In a mouse xenograft model, FPS-ZM1 inhibited primary tumor growth, suppressed tumor angiogenesis and inflammatory cell recruitment, and prevented metastasis to the lung and liver compared to vector-treated controls (179).

### 5.2 Targeted RAGE intracellular segment inhibitors

Targeting the intracellular segment of RAGE and interfering with ctRAGE-DIAPH1 interaction is a novel mechanism to specifically inhibit RAGE signaling. Recently, Ann Marie Schmidt’s team has developed a small molecule inhibitor called RAGE229 that inhibits the interaction between ctRAGE and DIAPH1, thereby completely inhibiting signaling within RAGE cells (205). In a diabetic mouse model, RAGE229 attenuated short-term and long-term diabetic complications in both male and female mice without reducing blood glucose concentrations. RAGE229 treatment reduced plasma concentrations of TNF-α, IL-6 and CCL2/JE-MCP1 in diabetic mice, while reducing pathological and functional indicators of diabetic-like nephropathy. As a novel small molecule inhibitor targeting protein interactions, RAGE229 is expected to be used for the treatment of short-term and long-term complications caused by diabetes.

## 6 Summary and prospect

RAGE is expressed at low levels under normal physiological conditions, but under chronic and persistent inflammatory conditions, RAGE signaling is upregulated and involved in the development of multiple diseases. Targeting RAGE appears to be a promising therapeutic strategy to control RAGE-mediated diseases. Several approaches to block RAGE signaling have been proposed and are being investigated, however, most are in preclinical studies and further human clinical trials need to be implemented to validate their safety and efficacy for the treatment of multiple RAGE-related diseases. In addition, because RAGE also has an important role under normal physiological conditions, future studies need to have a better understanding of the advantages and disadvantages of RAGE-targeted therapies and the long-term effects of RAGE blockade in humans before possible targeted RAGE therapeutic strategies can be established.

## Author contributions

HaD contributed to conception, design and drafted the manuscript. YZ collected the date and designed the figures. YH performed literature search, and provided valuable comments. HuD performed manuscript review and final version approval. All authors contributed to the article and approved the submitted version.

## Acknowledgments

We wish to thank Figdraw platform for providing the graphic materials.

## Conflict of interest

The authors declare that the research was conducted in the absence of any commercial or financial relationships that could be construed as a potential conflict of interest.

## Publisher’s note

All claims expressed in this article are solely those of the authors and do not necessarily represent those of their affiliated organizations, or those of the publisher, the editors and the reviewers. Any product that may be evaluated in this article, or claim that may be made by its manufacturer, is not guaranteed or endorsed by the publisher.
